# A load forecasting method based on edge graph attention network

**DOI:** 10.1371/journal.pone.0326709

**Published:** 2026-04-23

**Authors:** Mengze Gu, Xueping Li, Yao Cai

**Affiliations:** Hebei Key Laboratory of Power Electronics for Energy Conservation and Drive Control, Yanshan University, Qinhuangdao, Hebei, China; National University of Defense Technology, CHINA

## Abstract

Given the increasing demand for high-accuracy power load forecasting, traditional load forecasting methods can capture long-term dependencies in time series, but cannot fully capture the complex relationships between multi-dimensional features. This paper proposes an innovative method to convert time series data into graph features. By constructing a graph structure based on time nodes, the time series forecasting problem is transformed into a graph-based load forecasting problem. On this basis, the Edge Graph Attention Network (EGAT) is used to combine the feature information of nodes and edges to further enhance the ability to represent feature interactions and improve the accuracy of load forecasting. This paper compares the EGAT model with common load forecasting methods, including gated recurrent units (GRU), multi-layer perceptron networks (MLP) and long short-term memory (LSTM). The results show that EGAT is effective at finding important features and understanding complex time patterns, which means it shows strong potential in predicting energy demand. A limitation of the proposed approach is its increased computational cost introduced by graph construction and attention-based aggregation, which may raise training time and memory usage for large-scale graphs. In addition, the forecasting performance can be influenced by the design of the time-series graph (e.g., connectivity patterns) and the availability/quality of edge features.

## 1. Introduction

With the growth of energy networks and smart power systems, the operation and management of the power system has gradually shifted from past experience-based decision-making to relying on accurate data-driven prediction. This means that load forecasting needs to be more accurate. Accurate short-term load forecasting can provide reliable data support for the power grid dispatching center, ensure the dynamic balance of power supply and demand, and thus ensure the safe and stable operation of the power grid. Ref. [1] emphasizes the revolutionary impact of machine learning (ML) and artificial intelligence (AI) on load forecasting. With the increase of smart meter data, weather data and other power grid-related variables, the load forecasting model needs to consider various influencing factors (such as weather changes, historical load data, etc.) to reduce prediction errors and improve system efficiency [2,3]. Time series load forecasting is typically split into two parts: long-term load forecasting and short-term load forecasting. Long-term load forecasting is usually for a period of one year or longer, while short-term load forecasting is typically for a few days to one week. Ref. [4,5] emphasizes the importance of long-term and short-term forecasting to power system planning, which helps to achieve sustainable development of the power system. In power grid dispatching, every 1% improvement in prediction accuracy may significantly reduce power generation costs (about 0.1% to 0.3%) and reduce carbon emissions [6].

In short-term load forecasting, time series data contain rich temporal information. RNNs, designed for sequential data, can grasp connections within it, aiding in understanding sequence complexity [7,8]. However, regular RNNs often face gradient vanishing or exploding issues with long sequences, limiting performance [9]. To address this issue, GRU and LSTM (long short-term memory) models are commonly used to predict future values from historical time series data [10]. Ref. [11] uses LSTM and GRU models to predict peak load by incorporating factors like temperature extremes and load data from several years. Ref. [12] compares GRU and LSTM in short-term load forecasting, finding GRU has better MAPE performance and handles nonlinear features well [13]. MLP, a static feedforward network, is used for independent data prediction and performs well in net load forecasting [14,15]. Ref. [16] proposes a TCN-MLP hybrid model that combines TCN’s time series feature extraction with MLP’s nonlinear mapping for improved prediction. Overall, LSTM and GRU excel in capturing time series features, while MLP also has advantages in handling nonlinear relationships [17].

Traditional time series prediction models struggle to capture complex dependencies and external influences, often directly modeling one-dimensional data and failing to utilize intricate relationships and multi-feature interactions. Ref. [18] introduced a method converting time series into topological graphs, transforming temporal processing into spatial processing to retain key data point relationships, thereby enhancing clustering performance [19]. This approach allows time series data to be converted into shape feature evolution graphs, which can be modeled using graph neural networks. Multiple attention mechanisms can then capture key features, effectively learning the time series [20].

Time series models can be transformed into spatial topological graphs, enabling the application of graph neural networks (GNNs) for tasks like node classification and link prediction. GNNs model graph-structured data and capture node relationships and topological information [21]. Ref. [22] proposed a GNN-based method for accurate fault location prediction, constructing a GNN model that integrates node and link attributes to model the distribution system topology. By learning graph structure features and combining topology information, fault locations can be identified. GNNs effectively generalize message passing mechanisms, updating node representations by summarizing neighbor node information, suitable for various graph data prediction tasks [23,24]. Ref. [25] proposed an efficient topology recognition method using GNN, modeling it as a graph edge prediction problem.

GNNs typically use fixed aggregation methods for feature extraction, which cannot flexibly adjust neighbor node importance. Ref. [26] proposes GAT, which dynamically assigns weights to neighbor nodes using attention, effectively capturing node importance differences and improving model performance, especially for large-scale graph data. However, GAT neglects edge information. Ref. [27] suggests utilizing edge features to enhance GNN performance. EGAT, an extension of GAT, incorporates edge features during node feature aggregation, jointly learning node and edge features to improve model prediction capabilities [28,29]. Compared with existing studies that convert time series into graph representations for downstream learning tasks, our work focuses on short-term load forecasting and constructs a time-series topology graph tailored to this setting. Specifically, we model the load values as node features and represent exogenous variables (e.g., temperature) as edge features, so that external influences can be explicitly incorporated during information propagation. In addition, unlike conventional GAT-based graph models that primarily aggregate node information, we adopt EGAT to jointly leverage node and edge features in the attention-based message passing process. This design enables the model to capture temporal adjacency, periodic dependencies, and multi-feature interactions in a unified graph framework for improved load forecasting.

The main points of this paper are:

1. Modeling traditional time series features based on graph theory, converting time series into topological graphs. The graph attention mechanism combines information from both the nodes and edges to better understand the complex structure of the graph. This helps to improve prediction accuracy by using the different features of nodes and edges together.
2. The model is compared to the traditional time series prediction models, GRU, LSTM and MLP. Experiments show that EGAT is better than other prediction models in single-step time series load prediction accuracy.
3. An ablation study is conducted to analyze the contributions of edge features and the multi-head attention mechanism. Experiments show that the complete EGAT model achieves better forecasting performance than its simplified variants.

## 2. Load forecasting model

To better model multi-dimensional influences in short-term load forecasting, this paper follows a graph-based forecasting pipeline. Section 2.1 briefly summarizes the conventional time-series forecasting setting, while Section 2.2 converts the multivariate time series into a time-series load graph (Fig 2), where load is represented as the node feature and other related variables are encoded as edge features. Based on these graph samples, EGAT jointly aggregates node–edge information via attention to produce load predictions (Fig 3), which are optimized against the ground truth.

### 2.1. Traditional load forecasting model

This subsection describes the conventional time-series forecasting setting, including the input–output structure and single-/multi-step tasks.

**Fig 1:**
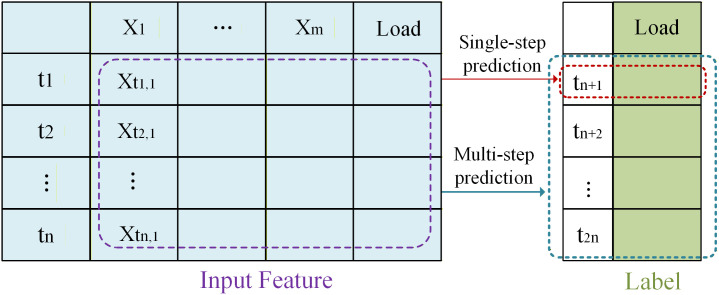
Traditional load forecasting model based on multi-dimensional time series data, where historical features are used to predict future load values.

Traditional load forecasting predicts how much electricity will be needed in the future by looking at past usage data, weather conditions, economic growth, and other influences. Traditional load forecasting, such as GRU, is based on time series and predicts by mining the trend, periodicity and random fluctuations of historical data. Load forecasting can be grouped into three types based on the time frame: short-term, medium-term, and long-term. As shown in Fig 1, the traditional time series prediction, the table shows the traditional time series feature structure, each moment contains multi-dimensional features. Load is the main feature, *X1* to *Xm* are weather conditions and other factors. In short-term prediction, we use all the feature data over time as input and the load as the output we want to predict. The traditional load forecasting model uses data to understand and learn. Prediction can be split into two types: single-step prediction and multi-step prediction. Single-step is to use the data from *1* to *n* to predict the load at the *n + 1* moment, and multi-step is to use the data from *1* to *n* to predict all loads from *n + 1* to *2n*.

Traditional time series prediction models have certain limitations in describing complex temporal dependencies and external environmental influences. These models usually directly use one-dimensional time series for modeling, which makes it difficult to fully explore the complex relationships between data points and the interactions between multiple features. This paper improves on the traditional prediction structure, converts ordinary time series into graphs, and on this basis represents the feature data in the graph to obtain a basic graph prediction model.

### 2.2. Design of time-series load graph model

This subsection describes the construction of the time-series load graph, including node and edge definitions and topology design.

**Fig 2:**
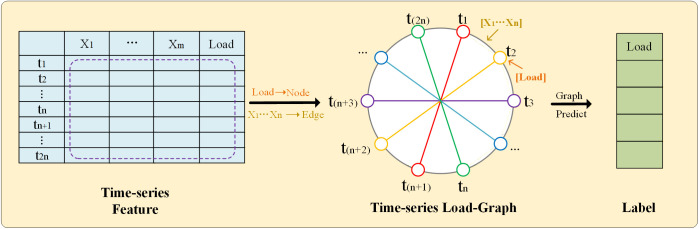
Time-series Load Graph Model, where node features represent load values and edge features represent other related variables.

In Fig 1, based on the traditional time series structure, the time series feature table is converted into a graph, and the previous time series feature input is changed to a graph feature input. In order to better establish the load forecasting graph model, the expression of the number of moments is changed based on Fig 1, because the number of moments in a day is generally an even number, as shown in Fig 2, we change the number of moments from *n* to *2n*. Then the time series nodes are connected to form a topological graph containing edges and nodes. Specifically, each time step in the time-series window is treated as a node in the graph. Let the length of the input time window be *2n*. Then the constructed graph contains *2n* nodes, denoted as *V* = {*v*_*1*_, *v*_*2*_, …, *v*_*2n*_}, where *v*_t_ corresponds to the *t*-th time moment in the time series. In the time series graph, the load is represented as a node feature on each node in the graph, and other features are represented as edge features on each edge in the graph. In this study, exogenous variables such as dry bulb temperature,wet bulb temperature, and dew point temperature are used as edge features. For an edge connecting node *v*_i_ to node *v*_*j*_, the edge feature vector is constructed from the external variables at time *t*. After forming the topological graph, the time nodes are abstractly extracted, and the feature representation is equivalent to the conversion from the original time series feature to the graph feature. In this way, we can use the characteristics of the graph to connect the time nodes through the graph attention mechanism. The purpose of this design is to capture the mutual influence between the previous and next nodes and enhance the propagation of time series information between different nodes. In the process of converting the time series into a topological graph, it is necessary to consider the representation of nodes and edges in the topological graph. We assign load features to the nodes in the graph and other features to the edges, thereby highlighting the importance of the load feature.

Two types of edges are constructed in the time-series load graph.

1) Temporal adjacency edges.

Each node *v*_*t*_ is connected to its adjacent time step *v*_*t+1*_ to preserve the temporal dependency in the original time series.

2) Periodic connection edges.

To capture the periodic characteristics of electricity load, additional edges are constructed between nodes separated by *n* time steps. Specifically, *v*_t_ is connected to *v*_(*t+n*)_, such as the connection between *t*_*1*_ and *t*_(*n+1*)_shown in Fig 2.

Through these two types of edges, the graph structure can capture both short-term temporal relationships and long-term periodic dependencies in the load data.

The total number of edges is composed of two parts: n temporal adjacency edges and *2n* periodic connection edges used to enhance graph connectivity, resulting in a total of *3n* edges.,

This method extracts load data as node features and other features as edge features, effectively aggregates the time series topology structure, and ensures the accuracy of feature information. On this basis, the matching labels are generated for subsequent model training.

## 3. Edge graph attention forecasting model

### 3.1. Method of load forecasting

This subsection describes the overall EGAT-based forecasting framework.

**Fig 3:**
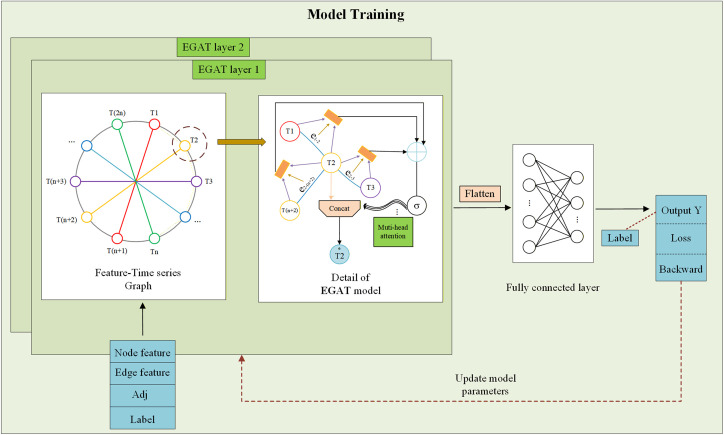
Training framework of the EGAT-based load forecasting model, including graph-based feature extraction and load prediction.

Since the time series topology graph can form a complicated network, choosing and using its node and edge features is important for making load predictions more accurate. This paper suggests a way to make predictions using a method that focuses on an edge-graph attention technique, as shown in Fig 3. Through the analysis of the load time series graph, the model can have a high accuracy in predicting load.

The fundamental prediction model consists of a feature input layer, a graph attention mechanism for feature extraction, a fully connected layer, and an output layer. The input layer uses time series data and combines the characteristics of both nodes and edges in the graph to understand the graph’s shape and connections. After sample processing, the node and edge features, along with their respective dimensions, are fed into the EGAT model. This model uses a multi-layer to extract intricate feature. In the feature fusion step, the EGAT model uses a multi-head attention method. By working with several attention patterns at the same time, the model gets much better at noticing important information, thereby improving its representational capacity and learning efficiency. After feature fusion, the fused node and edge features are input to the fully connected layer for prediction. The prediction is compared with the actual value to compute the loss, and the model’s settings are adjusted using backpropagation to improve how accurate the predictions are. The EGAT model’s capability for time series load prediction is then evaluated to verify the accuracy of its load predictions.

### 3.2. Model input

This subsection defines the model input, including node features, edge features, and the corresponding edge index matrix.

The EGAT model uses a graph to show its input features. This graph has three main parts: the characteristics of the nodes, the characteristics of the edges, and the matrices that index the edges. In simple words, the graph is explained like this:


$$G=\left(A,H^{\text{Load}},E\right)$$ (1)

where *A* represents the edge index matrix; *H* ^Load^ represents the list of features for nodes; *E* represents the list of features for edges.

The input includes the topology structure, node data, and edge data. Load is used as the node feature, dry bulb temperature, wet bulb temperature, and dew point temperature are used as edge features, and load is used as the label.

The load time series graph shows how the connections between nodes are represented using the edge index matrix *A*. Each row [*i*, *j*] in the matrix represents the edge between the considered node *i* and node *j*. The matrix *A* as a whole represents the nodes connected sequentially and then connected diagonally, for example, *n*—2*n* represents a diagonal in the graph.


$$A=\left[\begin{array}{c} @l@1\;2\\ 2\;3\\ \text{ \textbackslash{}ldots}\\ \text{2n-}1\;2\text{n}\\ \text{2n }\;1\\ 1\;\;\;\text{n+1}\\ \text{2}\;\;\;\text{n+}2\\ \text{ \textbackslash{}ldots}\\ \text{n }\;\;2\text{n} \end{array}\right]$$ (2)

Then, all feature data are adjusted using the minimum-maximum method. The data is handled using a moving window. Each moving window uses data from *2n* moments to make single-step predictions and multi-step predictions, predicting the node features of the next moment, that is, the *2n + 1* moment, and the node features of the *2n + 1* to *4n* moments.

Load is used as node feature, and the node feature set is as follows:


$$H^{\text{Load}}=\left\{\overset{\text{Load}}{\underset{\text{1}}{H}},\overset{\text{Load}}{\underset{\text{2}}{H}}\text{,}\cdots \text{,}\overset{\text{Load}}{\underset{f}{H}}\right\}$$ (3)

Where $\overset{L\text{oad}}{\underset{s}{H}}$ is the node load value at the *s-th* moment (*s* = *1*,*2*,…  , *f*, *f* denotes the number of time steps.).

The edge feature data set is as follows:


$$E=\left\{E^{\text{1}},E^{\text{2}}\text{,}\cdots ,E^{f}\right\}$$ (4)

Among them:


$$E^{t}=\left\{\overset{t}{\underset{\text{1}}{E}},\overset{t}{\underset{\text{2}}{E}}\text{,}\cdots ,\overset{t}{\underset{p}{E}}\right\}$$ (5)

where *t* = *1*,*2*,…, *f*, *f* is the number of moments. $\overset{t}{\underset{q}{E}}$ is the eigenvalue of the *q-th* (*q* = *1*,*2*,…,*p, p* is the total number of features) feature at the *t-th* moment.

### 3.3. Multi-head graph attention mechanism

EGAT is a sophisticated type of GNN tailored for handling edge features, addressing the shortcomings of conventional GNN frameworks that primarily focus on node features and often oversimplify the processing of edge information. By incorporating an edge graph attention mechanism, EGAT can dynamically concentrate on crucial edge information, thereby enhancing its ability to capture complex relationships between nodes and effectively utilize edge features. This capability is especially significant in domains like power grid topology analysis, where precise modeling and thorough exploitation of edge features are essential for improving analysis accuracy and predictive performance.

The core of EGAT is to calculate attention scores and node aggregation.The model used in this article is GATv2. Unlike GAT, the weight vector is placed before LeakyReLU and a dynamic attention mechanism is used, which means that it depends not only on node features but also on adjacency. The attention weight is normalized as follows:


$$\alpha_{ij}=\frac{exp\left(W^{T}\text{LeakyReLU}\left(\left[W^{\alpha }x_{i}∥W^{\beta }x_{j}∥W^{\gamma }e_{ij}\right]\right)\right)}{\sum_{k\in N_{i}}exp\left(W^{T}\text{LeakyReLU}\left(\left[W^{\alpha }x_{i}∥W^{\beta }x_{k}∥W^{\gamma }e_{ik}\right]\right)\right)}$$ (6)

Where exp refers to the exponential function operation, $x_{i}$, $x_{j}$, $x_{k}$ represents the feature vector of node i, j, k, $e_{ij}$ and $e_{ik}$ represent the edge feature vectors of edges (i,j) and (i,k) respectively, ∥ represents the vector connection operation, $N_{i}$ is the set of the first-order neighborhood of node i and node i itself, $W^{T}$ represents the transpose of the weight vector for parameterization, and the learnable parameters $W^{\alpha }$, $W^{\beta }$, $W^{\gamma }$ are used to linearly transform the node and edge features respectively. The nonlinear activation function is LeakyReLU. The expression of LeakyReLU is as follows:


$$\text{LeakyReLu(x)}=\left\{ \begin{array}{c} @c@x,x>0\\ kx,x\leq 0 \end{array}\right.$$ (7)

The weighted sum is calculated using a nonlinear activation function σ and is used as the output of the node attention module, which can be expressed as:


$$\overset{\prime }{\underset{i}{x}}=\sigma (\sum_{j\in N_{i}}\alpha_{ij}W^{\delta }x_{j}+\sum_{j\in N_{i}}\alpha_{ij}W^{\eta }e_{ij})∥x_{i}$$ (8)

Where $\overset{\prime }{\underset{i}{x}}$ represents the updated node feature i vector, $W^{\delta }$, $W^{\eta }$ is the learnable update parameter, and the output after activation is concatenated with $x_{i}$ to generate a new feature.

Due to the complex topological structure of time series, it is difficult for information to be captured by general feature aggregation methods. So, a multi-head attention method is used to find the attention weight by looking at how similar the target node is to the edge. It checks how similar the nodes are and turns that into the attention weight. The main idea of the multi-head attention mechanism is to break down one attention process into several separate attention heads. Each head works on its own to figure out the attention weight. The results from these attention heads are combined to create a clearer and more useful set of features. The connection between the features of nodes and the features of edges is created using the multi-head attention method:


$${\alpha_{ij}}^{\left(m\right)}=\frac{exp\left(W^{T\left(m\right)}\text{LeakyReLU}\left(\left[W^{\alpha \left(m\right)}x_{i}∥W^{\beta \left(m\right)}x_{j}∥W^{\gamma \left(m\right)}e_{ij}\right]\right)\right)}{\sum_{k\in N_{i}}exp\left(W^{T\left(m\right)}\text{LeakyReLU}\left(\left[W^{\alpha \left(m\right)}x_{i}∥W^{\beta \left(m\right)}x_{k}∥W^{\gamma \left(m\right)}e_{ik}\right]\right)\right)}$$ (9)

Among them, ${\alpha_{ij}}^{\left(m\right)}$ represents the weight of node *j* to node *i* in the *m*th attention head. $\overset{\delta }{\underset{node}{W}}$, $\overset{\eta }{\underset{edge}{W}}$ is the weight matrix, which is used for node feature and edge feature update respectively. The output of different attention heads is:


$${\overset{\prime }{\underset{i}{x}}}^{\left(m\right)}=\sigma (\sum_{j\in N_{i}}{\alpha_{ij}}^{\left(m\right)}\overset{\delta }{\underset{node}{W}}x_{j}+\sum_{j\in N_{i}}{\alpha_{ij}}^{\left(m\right)}\overset{\eta }{\underset{edge}{W}}e_{ij})∥x_{i}$$ (10)

Average the output of all attention heads and finally get the representation of the node *i*:


$$\vec{\overset{\prime }{\underset{i}{x}}}=\frac{\text{1}}{P}\sum_{m=1}^{P}{\overset{\prime }{\underset{i}{x}}}^{\left(m\right)}$$ (11)

There are a total of *P* attention heads, and *m* represents the *m*th attention head.

### 3.4. Model output

This subsection presents the output layer and loss function used for load forecasting.

The model output is the node feature $\vec{\overset{\prime }{\underset{i}{x}}}$ after EGAT aggregation. The output is first flattened by Flatten to obtain $\overset{final}{\underset{i}{x}}$, and then the node features are output through the fully connected layer to get the prediction result $\mathrm{\backslash stackrel}\wedge y_{i}$.


$$\overset{final}{\underset{i}{x}}=flatten\left(\vec{\overset{\prime }{\underset{i}{x}}}\right)$$ (12)

$$\mathrm{\backslash stackrel}\wedge y_{i}=FC\left(\overset{final}{\underset{i}{x}}\right)$$ (13)

Throughout the training process, labels are used to supervise learning and optimize the loss function. The loss function uses the mean squared error (MSE) to find out how far off the predicted value is from the real value.


$$MSE=\frac{\text{1}}{\text{n}}\sum {\left(x_{i}-y_{i}\right)}^{\text{2}}$$ (14)

Among them, *n* is the number of samples, $x_{i}$ is the true value of the *i*-th sample, and $y_{i}$ is the predicted value of the *i*-th sample.

## 4. Simulation analysis

### 4.1. Dataset processing

In order to verify the performance of the EGAT model in time series feature prediction, the load data of a region in New South Wales, Australia from 2006 to 2007 [30] were selected for training and prediction. The data was a time node every half an hour, and there were 48 time nodes in a day, generating a total of 35,040 sample moments. The prediction ability of the model was verified by introducing multidimensional data. In addition to load data as features, the data also included dry bulb temperature, wet bulb temperature, and dew point temperature. A total of four features were used to predict the load. According to different local temperature fluctuations, the correlation between temperature features and the load changes caused by them was extracted, and the training and learning was carried out by training the EGAT prediction model.

The data is divided into training set and test set. This paper uses the data of the whole year of 2006 for training and the data of the whole year of 2007 for testing.

In order to show the performance of graph prediction in different situations, there are two groups of data in total, one of which uses the original data, namely data-1. The other group extracts data in full hours and removes the half-hour data between two full hours, thus reducing the amount of data by half, which is data-2. The two groups of data are divided into two groups to verify the prediction effect of the graph prediction model on data sets with different complexities. Based on the two groups of data, the experiment is divided into three cases for simulation:

**Case 1**: Based on data-1, 24 hours a day, every half hour is a moment, the number of nodes input to the model is 48, and the number of edges is 72. Using two years of data, a total of 35040 samples are generated, 17520 samples in the first year are the training set, and 17520 samples in the second year are the test set. This case performs a single-step load forecasting simulation (using the first 48 moments to predict the next moment).

**Case 2**: In order to verify the different effects of the model on the effects of single-step prediction and multi-step prediction, this case performs a multi-step load forecasting simulation based on the same data as case 1 (using the first 48 moments to predict the next 48 moments).

**Case 3**: Based on data-2, 24 hours a day, every hour is a moment, the number of nodes input to the model is 24, and the number of edges is 36. Using two years of data, a total of 17,520 samples were generated. 8,760 samples in the first year were used as a training set, and 8,760 samples in the second year were used as a test set. In this case, a single-step load forecasting simulation experiment was conducted (using the first 24 moments to predict the next 1 moment).

This study used the MATLAB R2021 b software platform to process sample data. The experimental simulation was performed on a PC equipped with a 12th generation Intel (R) Core (TM) i7-12500 H processor. The simulation environment was Python 3.8 and PyTorch 2.1.0.

Among the prediction evaluation indicators, the determination coefficient R², mean absolute percentage error (MAPE), mean absolute error (MAE) and mean square error (MSE) are used as evaluation indicators. According to the indicator values and curves, the performance of different prediction methods is compared and analyzed.

### 4.2. Model training

In order to evaluate the performance of the proposed method and other neural network methods, the gated recurrent unit (GRU) model, the long short-term memory (LSTM) model, and the multi-layer perceptron (MLP) were used to conduct corresponding experiments to ensure consistent data division standards. GRU and LSTM can effectively capture time series information and have certain advantages in the prediction effect of simple time series features. Although MLP has a simple structure, it also has high accuracy and computational efficiency in prediction.

A total of three groups of simulation experiments were conducted, namely **Case 1**, **Case 2** and **Case 3**. Three models were used for comparison in each group, namely the graph prediction model EGAT, GRU and MLP, LSTM model, and the epoch was set to 300 to verify the load prediction effects of different models.

The EGAT model uses two edge graph attention layers and the number of attention heads is set to 4 to improve feature aggregation capabilities. The learning rate is set to 0.00008 to ensure stable convergence. The LeakyReLU activation function effectively alleviates the gradient vanishing problem by introducing a negative slope, maintains nonlinear characteristics, and accelerates convergence. The output layer is flattened to the fully connected layer after passing through the EGAT layer to achieve dimensionality conversion.

GRU, MLP and LSTM models use batch processing with a batch size of 32 and a learning rate of 0.0001 to ensure stable convergence. GRU and LSTM set two hidden layers with 64 neurons. MLP sets two linear layers with 32 and 16 neurons.

In terms of computational complexity, the EGAT model mainly consists of graph construction and attention-based message passing operations. Compared with traditional time-series models such as GRU and MLP, the graph attention mechanism introduces additional computations due to the attention weight calculation and feature aggregation across graph edges.

However, in this study the constructed time-series graph contains a limited number of nodes and edges (e.g., 48 nodes and 72 edges in Case 1), which keeps the computational cost manageable. Therefore, although the proposed EGAT model introduces additional computational overhead compared with conventional neural networks, the training time and computational requirements remain acceptable for practical short-term load forecasting applications.

### 4.3. Simulation results

In this section, the evaluation index results of various prediction methods for load forecasting under different samples are shown, the prediction effects of EGAT model, GRU, MLP and LSTM are comprehensively compared, and the advantages of the prediction method based on the edge-graph attention mechanism are analyzed.

After the model training is completed, the loss function converges, and the final saved training model is put into the test set for evaluation.

If R² is close to 1, it means the model fits well. Also, lower MSE, MAPE, and MAE values mean less error in the predictions.

#### 4.3.1. Case 1 analysis.

**Fig 4:**
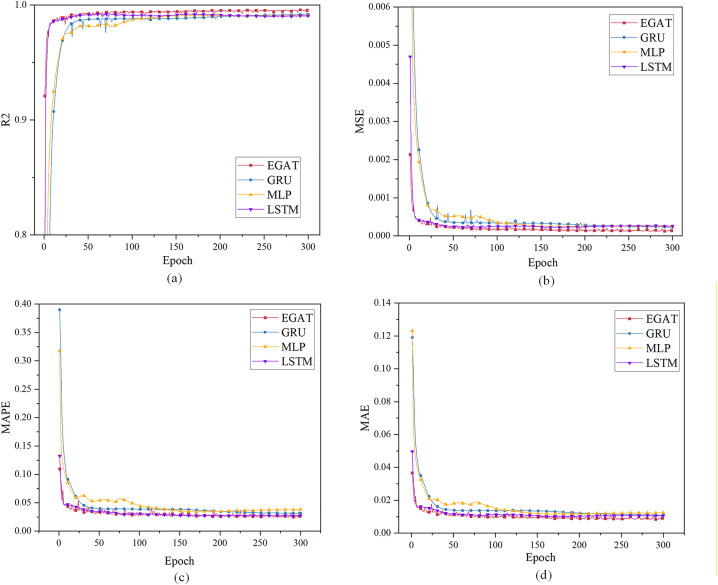
Single-step load forecasting performance comparison of different models in Case 1.

For case 1, using the load characteristic data of the previous 48 moments to predict the load data of the next moment. Fig 4 and Table 1 show the evaluation index results. All curves have converged when the epoch is 300. When the EPOCH of GRU and MLP is very small, R² is less than 0, therefore part of the curve is intercepted.

**Table 1:** Case 1 simulation results.

Indicator Method	R^2^(%)	MSE	MAPE	MAE
EGAT	99.59	0.00012	0.02469	0.0083
GRU	99.20	0.00023	0.0316	0.0109
MLP	99.09	0.00025	0.0377	0.0123
LSTM	99.11	0.00026	0.0273	0.0107

The Case 1 simulation results in Table 1 results show that the convergence R² value of EGAT reaches 99.59%, which is higher than the GRU, LSTM and the MLP model, and is about 0.39% higher than GRU, LSTM and MLP. This is due to the use of the connection between nodes in the topology graph, which allows nodes to notice more effective nodes in addition to adjacent nodes, making it easier for the model to capture data fluctuations. EGAT is lower than the others in the MSE, MAE, and MAPE evaluation systems. EGAT’s MSE is about 50% of the others, and MAPE and MAE are about 20% lower than GRU and LSTM, and about 30% lower than MLP. Because the EGAT model learns the correlation between node feature changes and edge feature changes, the prediction error performance is better and the overall prediction result is the most accurate.

**Fig 5:**
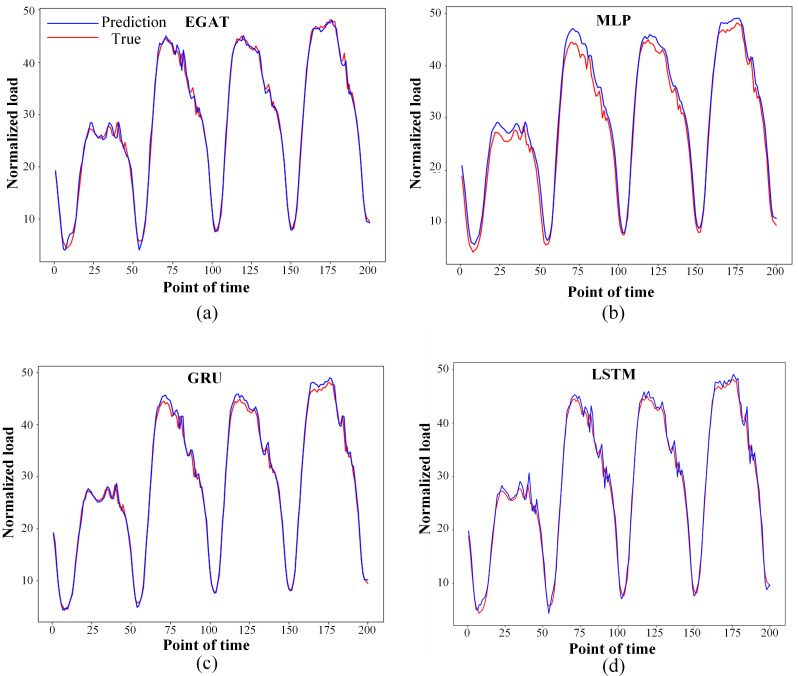
Fitting curves of predicted and actual load values on the test dataset.

The prediction effect is shown by selecting the fitting curve of the first 200 moments in the test data set, as shown in Fig 5. The ordinate is the normalized load value, the abscissa is the moment. The red line shows the real value, and the blue line shows the expected value. The fitting curve of the EGAT model shows that the prediction trend is basically consistent with the true value trend, and there is rarely any misfit in the curve. The prediction results of the MLP, GRU and LSTM methods fluctuate somewhat compared with the true value, and fluctuate greatly around the 75th, 125th and 175th time points. The prediction effect is average. This is because the GRU, MLP and LSTM models do not analyze the time series feature information enough, resulting in unreasonable predictions.

#### 4.3.2. Case 2 analysis.

**Fig 6:**
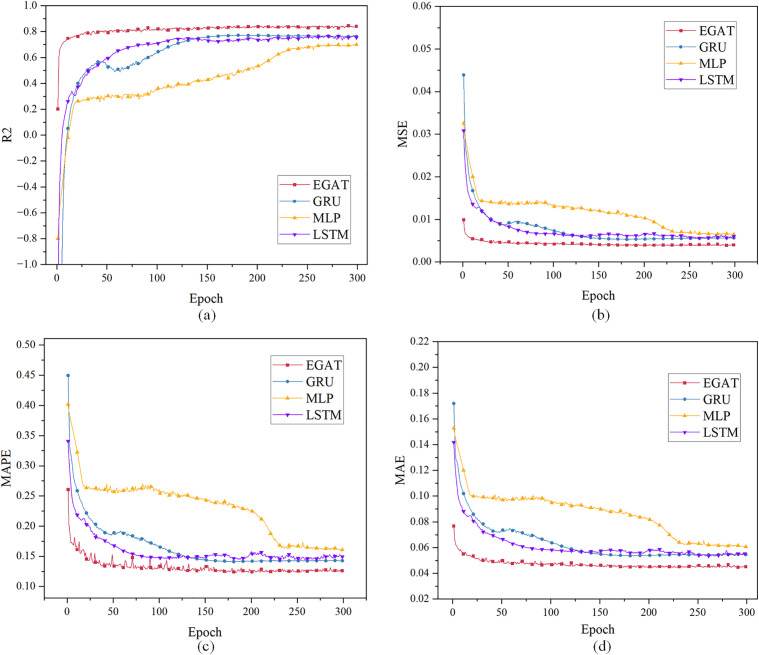
Multi-step load forecasting performance comparison of different models in Case 2.

Case 2: using the load characteristic data of the first 48 moments to predict the load data of the next 48 moments. Fig 6 and Table 2 show the prediction evaluation index results. When the epoch is 300, the curves have converged. When the epoch is less than 100, GRU, MLP and LSTM have very small R², while the EGAT model method converges faster, and the curve graph is partially intercepted.

**Table 2:** Case 2 simulation results.

Indicator Method	R^2^(%)	MSE	MAPE	MAE
EGAT	84.53	0.00391	0.1262	0.0446
GRU	77.21	0.00534	0.1413	0.0537
MLP	69.97	0.00651	0.1607	0.0608
LSTM	75.58	0.00583	0.1493	0.0552

The Case 2 simulation results in Table 2 results show that the EGAT model works much better than the GRU and MLP prediction models. The R² of EGAT reaches 84.53%, which is about 9% higher than the GRU model, about 20% higher than the MLP, about 12% higher than the LSTM. The MSE of the EGAT prediction model is about 25% lower than that of the GRU, and the MAPE and MAE are slightly lower than those of the others, because the error at certain specific moments is not as small as the overall error.

Single-step prediction is more accurate than multi-step prediction. Because of mistakes adding up, when making predictions over several steps, the model has to guess data for multiple time points, and the guess for each time point can rely on what it predicted before. Multi-step prediction must consider how data is related over a longer time. So, when predicting in several steps instead of just one, the two groups of EGAT models perform much better than the GRU and LSTM model. In EGAT single-step prediction, R² is 0.39% higher than the GRU model, and in multi-step prediction, R² is 9% higher than the GRU model. If the dynamic pattern of the data itself is complex, capturing these relationships requires stronger model capabilities, which can better reflect the differences between different models. This shows the excellent performance of the EGAT model in prediction.

**Fig 7:**
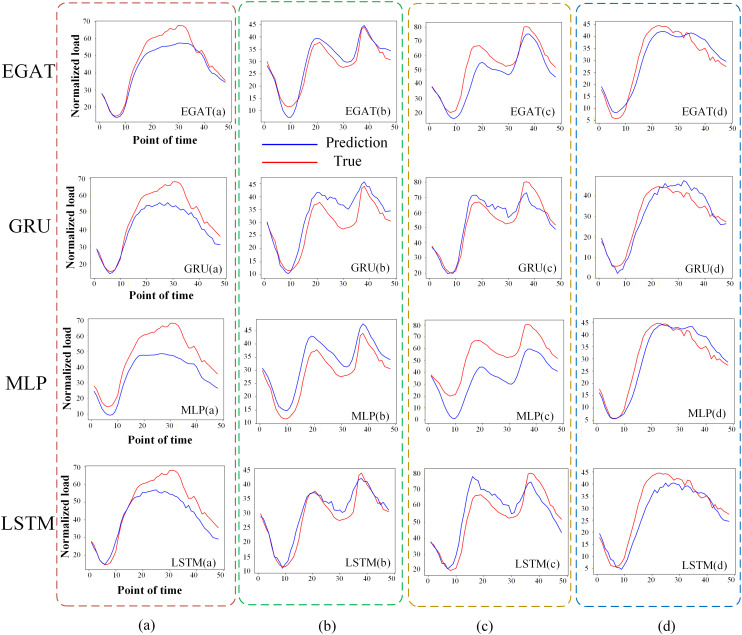
Load forecasting fitting curves for four typical days representing different seasons.

The typical time periods in four seasons (spring, summer, autumn and winter) were selected, corresponding to 48 moments in a day on March 1, June 1, September 1 and December 1, which correspond to (a), (b), (c) and (d) in the Fig 7, respectively, to fit the prediction effects of the three load forecasting models. In the (a) and (b) periods, the prediction error of EGAT in the second half of the day is small. It can be seen that the fitting effect of EGAT is significantly better than that of GRU, MLP and LSTM. In the (c) period, the curve of EGAT is the smoothest, while the fluctuation of GRU and LSTM are large and the error of MLP is too large. There is not much difference at the (d) moment. The analysis shows that the overall prediction effect of EGAT is significantly better than that of GRU, MLP and LSTM when the prediction test effect is fitted in four typical periods in a year.

#### 4.3.3. Case 3 analysis.

**Fig 8:**
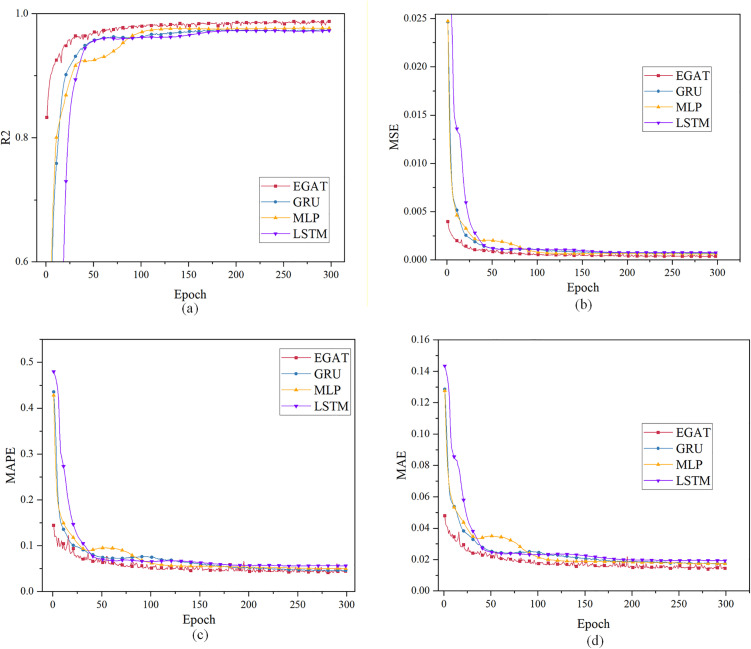
Single-step load forecasting performance comparison of different models in Case 3.

Case 3: Single-step prediction using the load characteristic data of the previous 24 moments to predict the load data of the next moment. Fig 8 and Table 3 show the evaluation index results. When the epoch is 300, all curves have converged. The results of the three prediction models are compared.

**Table 3:** Case 3 simulation results.

Indicator Method	R^2^(%)	MSE	MAPE	MAE
EGAT	98.72	0.00037	0.0422	0.0144
GRU	97.35	0.00071	0.0463	0.0175
MLP	97.66	0.00063	0.0505	0.0175
LSTM	97.26	0.00075	0.0562	0.0193

The Case 3 simulation results in Table 3 results show that in the 24-moment single-step prediction, the R²of the EGAT model reaches 98.72%, which is higher than the other prediction models. EGAT also performs better in other indicators.

Compared with the 48-moment single-step prediction in case1, the data used in the 24-moment single-step prediction in case3 is half less. Due to less data, the model’s ability to integrate and capture data information is more reflected. Compared with the 48-moment single-step prediction, the effect of the EGAT model in the 24-moment single-step prediction is greatly improved compared with the GRU model. In case1, the R² of EGAT is 0.39% higher than that of the GRU model, and in case3, the R² is 1.4% higher than that of the GRU model. When the data is sufficient, the prediction effect of GRU is close to that of EGAT, but in case3 with reduced data, the prediction effect of EGAT is significantly higher than that of GRU. This shows that when the data is reduced, EGAT can make full use of effective feature information, convert time information into spatial information for processing, better capture the connection between time series, and by adding effective edge information, it can better use the topological structure to analyze features, so as to more accurately predict the load.

### 4.4. Ablation study

To further investigate the effectiveness of different components in the proposed EGAT model, an ablation study is conducted. In particular, we analyze the contributions of the multi-head attention mechanism and the edge feature representation.

The ablation experiments are performed under the same experimental settings as the main experiments described in Section 4.2, including the same dataset, training–testing split, learning rate, and number of epochs. Three models are compared in the ablation study: the full EGAT model (**EGAT**), an EGAT model with single-head attention (**EGAT-SH**), and an EGAT model with no edge features (**EGAT-NE**).

For the single-head variant, the multi-head attention mechanism in EGAT is replaced by a single attention head while keeping other parameters unchanged. For the edge feature ablation experiment, all exogenous variables are moved to node features, while the edge features are set to zero. In this setting, the graph topology remains unchanged, but the model does not utilize edge feature information during message passing.

Three variants of the EGAT model are considered in this study:

**EGAT**: the full model with multi-head attention and edge features.

**EGAT-SH**: the single-head attention version of EGAT, used to evaluate the effect of the multi-head attention mechanism.

**EGAT-NE**: the model without edge features, where all variables are treated as node features and edge features are set to zero.

#### 4.4.1. Case 1 analysis(Ablation study).

**Fig 9:**
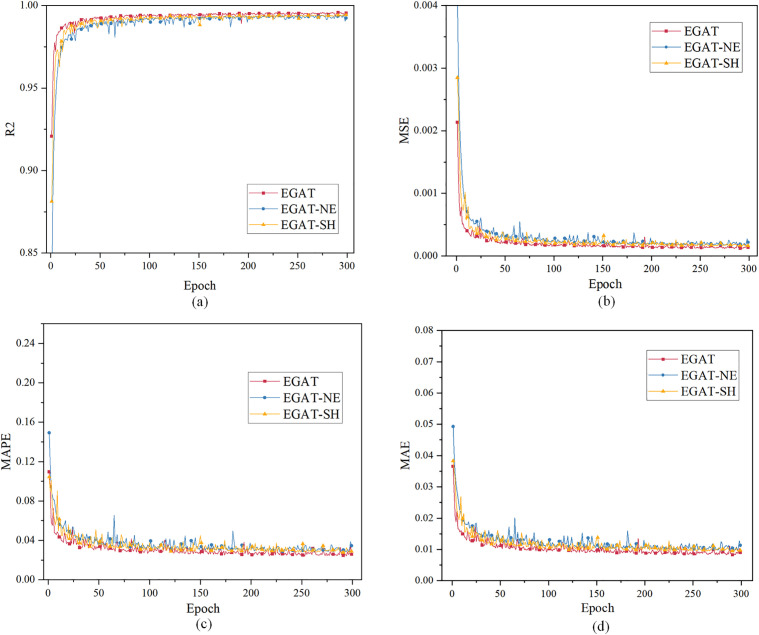
Single-step load forecasting performance comparison of different models in the ablation study (Case 1).

For case 1 in ablation study, using the load characteristic data of the previous 48 moments to predict the load data of the next moment. Fig 9 and Table 4 show the evaluation index results. All curves have converged when the epoch is 300. (**EGAT-SH:** EGAT with single-head attention. **EGAT-NE:** EGAT without edge features.)

**Table 4:** Case 1 ablation study results.

Indicator Method	R^2^(%)	MSE	MAPE	MAE
EGAT	99.59	0.00012	0.0247	0.0083
EGAT-NE	99.33	0.00017	0.0292	0.0099
EGAT-SH	99.45	0.00016	0.0277	0.0093

Table 4 presents the results of the ablation study for the proposed EGAT model under the 48-step single-step forecasting scenario. The full EGAT model achieves the best performance across all evaluation metrics.

When the edge features are removed (EGAT-NE), the prediction performance decreases noticeably. The R² value drops from 99.59% to 99.33%, while the error metrics (MSE, MAPE, and MAE) increase significantly. This indicates that modeling exogenous variables as edge features helps the model better capture the interactions between different time steps and improves the representation capability of the time-series load graph.

In addition, replacing the multi-head attention with a single-head attention (EGAT-SH) also leads to a performance degradation. Although the decrease is smaller compared with the edge-feature ablation, the results still demonstrate that the multi-head attention mechanism enhances the model’s ability to capture diverse feature relationships during the message-passing process.

#### 4.4.2. Case 2 analysis(Ablation study).

**Fig 10:**
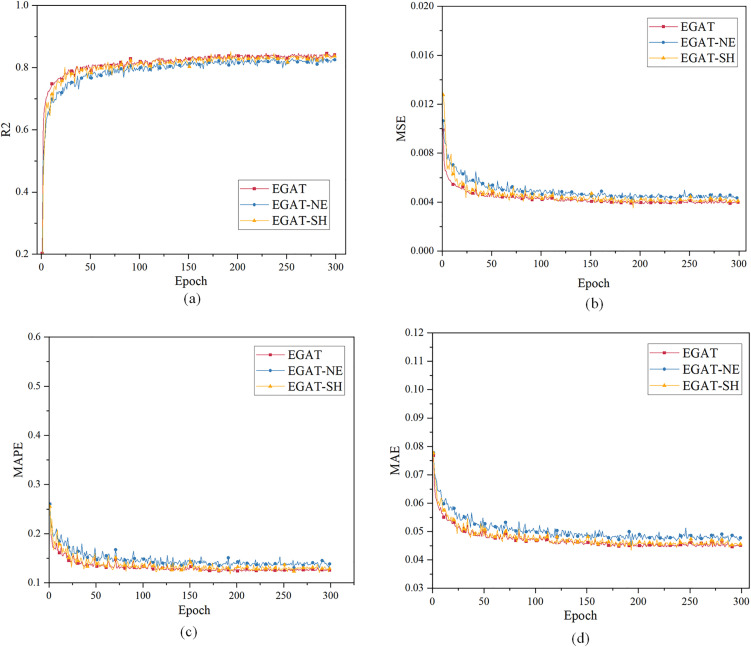
Muti-step load forecasting performance comparison of different models in the ablation study (Case 2).

For case 2 in ablation study, using the load characteristic data of the first 48 moments to predict the load data of the next 48 moments. Fig 10 and Table 5 show the evaluation index results. All curves have converged when the epoch is 300.

**Table 5:** Case 2 ablation study results.

Indicator Method	R^2^(%)	MSE	MAPE	MAE
EGAT	84.53	0.00391	0.1262	0.0446
EGAT-NE	82.62	0.00431	0.1411	0.0478
EGAT-SH	84.28	0.00413	0.1291	0.0456

As shown in Fig 10, all models converge stably during the training process. The proposed EGAT model consistently achieves the best performance across all evaluation metrics throughout the training process.

From Table 5, the full EGAT model obtains the highest R² value of 84.53% and the lowest error metrics, including MSE (0.00391), MAPE (0.1262), and MAE (0.0446). When the edge features are removed (EGAT-NE), the prediction performance decreases significantly. The R² value drops to 82.62%, and the error metrics increase accordingly. This indicates that modeling exogenous variables as edge features plays an important role in capturing temporal dependencies and improving prediction accuracy in multi-step load forecasting.

In addition, replacing the multi-head attention mechanism with a single-head attention (EGAT-SH) also leads to a slight performance degradation. Although the decrease is smaller compared with the edge-feature ablation, the results demonstrate that the multi-head attention mechanism helps the model capture richer feature interactions and enhances the representation capability of the graph structure.

#### 4.4.3. Case 3 analysis(Ablation study).

Case 3 in ablation study: Single-step prediction using the load characteristic data of the previous 24 moments to predict the load data of the next moment.

**Fig 11:**
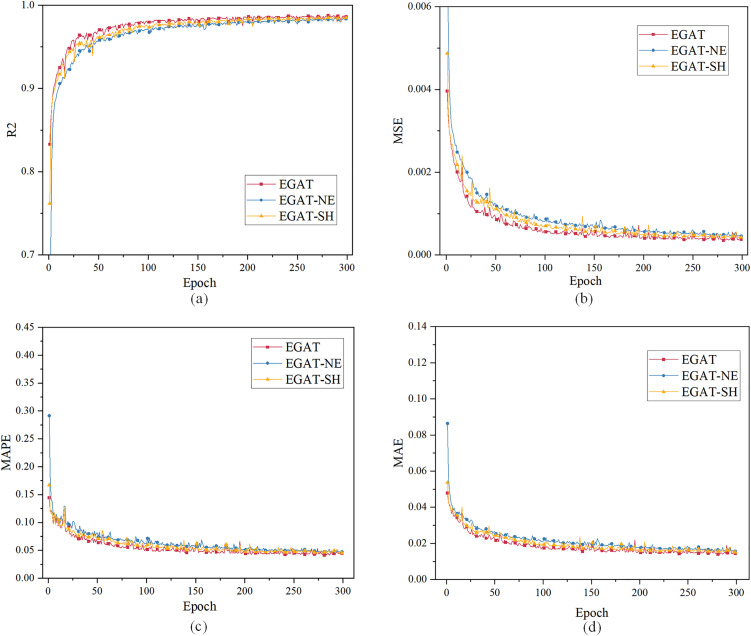
Single-step load forecasting performance comparison of different models in the ablation study (Case 3).

For Case 3, the ablation study is conducted under the single-step forecasting scenario using a reduced time window of 24 moments. In this setting, the load value of the next moment is predicted based on the previous 24 time steps. Fig 11 and Table 6 present the corresponding experimental results.

**Table 6:** Case 3 ablation study results.

Indicator Method	R^2^(%)	MSE	MAPE	MAE
EGAT	98.72	0.00037	0.0422	0.0144
EGAT-NE	98.38	0.00049	0.0473	0.0161
EGAT-SH	98.55	0.00043	0.0458	0.0155

All models converge stably during the training process, and the performance curves gradually stabilize after approximately 200 epochs. The proposed EGAT model consistently achieves the best performance among the three models across all evaluation metrics.

From Table 6, the full EGAT model obtains the highest R² value of 98.72% and the lowest prediction errors, including MSE (0.00037), MAPE (0.0422), and MAE (0.0144). When the edge features are removed (EGAT-NE), the prediction accuracy decreases noticeably, with the R² dropping to 98.38% and all error metrics increasing. This result indicates that modeling exogenous variables as edge features helps the model capture additional relationships between time steps and improves forecasting performance.

The superiority of the EGAT model is still maintained even when the input time window is reduced, demonstrating that the proposed graph-based representation can effectively utilize limited temporal information.

### 4.5. Discussion

The experimental results in Case 1–Case 3 consistently demonstrate that the proposed EGAT model achieves the best forecasting performance compared with GRU, LSTM, and MLP. The advantage of EGAT is especially evident in the multi-step forecasting scenario (Case 2), where error accumulation makes long-horizon prediction more challenging. This suggests that transforming time series into a graph and performing message passing across connected time nodes can capture richer temporal dependencies than conventional sequence models.

The ablation study further explains the performance gains. Removing edge features (EGAT-NE) causes a larger degradation than replacing multi-head attention with single-head attention (EGAT- SH), indicating that explicitly modeling exogenous variables (e.g., temperature) as edge attributes is a key factor in improving accuracy. Meanwhile, the multi-head attention mechanism provides additional but smaller improvements by learning diverse attention patterns during aggregation.

Despite the strong results, EGAT introduces additional computational cost due to attention-based aggregation over graph edges, which may increase training time compared with simpler baselines.

In addition, the current study is validated on a single regional dataset. Although the dataset is derived from a real-world electricity load dataset and reflects realistic load patterns, evaluating the proposed method on additional datasets from different regions and time periods will be an important direction for future work to further assess its generalization capability.

## 5. Conclusion

Although traditional load forecasting methods (such as GRU, LSTM and MLP) can capture the temporal characteristics of time series, they have deficiencies in complex multidimensional feature modeling and nonlinear feature interaction. To solve these problems, this paper proposes an innovative method to perform load forecasting by converting time series data into a time series topological graph and using the edge graph attention mechanism (EGAT). This method combines time nodes, edge features and adjacency relationships, and uses the graph attention mechanism to achieve interaction and aggregation of complex features, thereby improving prediction accuracy.

The main contributions of this study are threefold: (i) transforming multivariate load time series into a time-series topological graph for graph-based forecasting; (ii) representing load as node features and exogenous factors as edge features to explicitly model multi-feature interactions; and (iii) adopting a multi-head EGAT mechanism to jointly aggregate node–edge information and dynamically assign attention weights, addressing the limitations of conventional time-series models in multi-dimensional feature modeling.

Experiments demonstrate that EGAT achieves superior performance compared with representative baselines, including GRU, LSTM, and MLP. The advantage is consistent in single-step prediction and becomes more pronounced in the multi-step setting, indicating that the proposed graph representation and attention-based aggregation are effective for long-horizon forecasting where errors tend to accumulate. Moreover, EGAT maintains strong performance when the input window is reduced, suggesting that the graph-based formulation can exploit limited temporal information efficiently.

Ablation results confirm that explicitly modeling exogenous variables as edge features contributes most to the performance gains, while multi-head attention provides additional improvements. Future work will evaluate the method on more datasets and regions, explore stronger baselines such as Transformer-based models, and further optimize computational efficiency for large-scale deployment.

## Supporting information

S1 Data Minimal dataset underlying the results reported in this study. (XLSX)
